# Gut Microbe *Rikenellaceae_RC9_gut_group* and *Knoellia*-Mediated Acetic Acid Regulates Glucose and Lipid Metabolism in the Muscle of Freshwater Drum (*Aplodinotus grunniens*) Under High-Fat Diets

**DOI:** 10.1155/anu/9667909

**Published:** 2025-04-16

**Authors:** Miaomiao Xue, Pao Xu, Haibo Wen, Jiyan He, Jianxiang Chen, Changxin Kong, Xiaowei Li, Hang Wang, Xinxin Guo, Yi Su, Hongxia Li, Changyou Song

**Affiliations:** ^1^Wuxi Fisheries College, Nanjing Agricultural University, Wuxi 214081, China; ^2^Key Laboratory of Freshwater Fisheries and Germplasm Resources Utilization, Ministry of Agriculture and Rural Affairs, Freshwater Fisheries Research Center, Chinese Academy of Fishery Sciences, Wuxi 214081, China

**Keywords:** acetic acid, *Aplodinotus grunniens*, glycolipid metabolism, gut microbes, high-fat diet, muscle

## Abstract

Metabolic disorders and complications induced by high-fat diets (HFDs) are a hot research topic in aquatic animal nutrition and health, but the mechanism of gut microbes and their metabolites on muscle homeostasis is not yet clear. In this study, a 16-week HFD (Con, 6% fat and HFD, 12% fat) rearing experiment was conducted with a freshwater drum (20.88 ± 2.75 g, about 20,000 fish per pond) to investigate the underlying regulation of gut microbes on muscle nutrient and metabolism. Results revealed that HFD had no remarkable effect on proximate nutrients (moisture, ash, crude protein, and crude fat), total amino acids, and fatty acids contents in muscle. Moreover, decreased acetic acid content by HFD in the gut and muscle was confirmed to regulate lipid metabolism, as evidenced by the activation of fatty acid synthesis (acetyl-CoA carboxylase alpha [ACC1] and sterol regulatory element binding protein-1 [SREBP1]) and inhibition of fatty acid lipolysis (AMP-activated protein kinase [AMPK], adipose triglyceride lipase [ATGL], and carnitine palmitoyl transferase 2 [CPT2]). Interestingly, RNA-seq revealed glycolytic metabolism (glycolysis/gluconeogenesis and pyruvate metabolism) was active in the muscle under HFD, which was further confirmed to be the intermediate for acetic acid to regulate lipid metabolism. Strikingly, gut microbe *Rikenellaceae_RC9_gut_group* and *Knoellia* regulate muscle lipid and glucose metabolism through their derived metabolite acetic acid, which is the key target for gut microbe to regulate muscle. Taken together, these results reveal the regulatory mechanism of gut microbes and derived metabolites on muscle metabolism and development, providing a theoretical basis for the healthy regulation of HFD in aquatic animals.

**Summary**

• High-fat diets (HFDs) reduce acetic acid content in the intestine and muscle.
• Acetic acid is involved in intestinal and muscular glucose and lipid metabolism.
• *Rikenellaceae_RC9_gut_group* and *Knoellia* may be key targets in regulating acetic acid metabolism.
• *Rikenellaceae_RC9_gut_group* and *Knoellia* contribute to muscular glucose and lipid metabolism.

## 1. Introduction

With the rapid development of the social economy, people's need for aquaculture products is increasing [1]. However, the ever-increasing shortage of protein resources has a profound impact on aquaculture [2]. To conserve protein, high-fat diets (HFDs) are widely used in intensive aquaculture to improve growth performance and protein utilization [3, 4]. However, excess lipids intake inevitably leads to metabolic disorders, oxidative stress, lipid deposition, decreased immunity, impaired gut barrier, and reduced resistance to the environment in farmed fish [5–8]. Currently, HFD-induced metabolic disorders and syndromes have become a hot research topic in aquatic animal nutrition and health. The intestinal [9, 10] as important metabolic organs, have been of particular interest. However, the effects of HFD on gut microbes and their metabolites have not been sufficiently emphasized.

Muscle tissue accounts for the largest proportion of fish carcasses, thereby, is the main edible part of fish that provides high-quality protein and large amounts of essential amino acids (EAAs) and polyunsaturated fatty acids [11]. It is well known that the sources and levels of various nutrients, such as dietary protein, lipids, and carbohydrates, can affect muscle quality and metabolic capacity [12]. Among these, lipids are an essential energy source for muscle and determine muscle nutrient composition and metabolic homeostasis. However, numerous studies have shown that long-term HFD can cause muscle fat deposition, induce inflammatory responses, reduce antioxidant capacity, and even affect muscle nutrient composition and reduce muscle quality [13–15]. Given the dual effects of lipids, moderate quantity and quality of dietary lipids is particularly important to improve muscle metabolism, nutritional value, and muscle quality in aquaculture.

Research has shown that dietary lipids regulate gut bacterial composition and metabolic capacity [16], which further regulate host health and nutrient metabolism through a variety of mechanisms [17]. One of the important pathways is the involvement of gut microbe-derived metabolites of short-chain fatty acids (SCFAs) [18]. As products of anaerobic metabolism by intestine microbes of aquatic animals, SCFAs (including acetic acid, propionic acid, butyric acid, etc.) have been evidenced to be involved in energy metabolism [19], growth [20], immunity [21], and intestinal health [17]. Previous studies have found dietary acetic acid can improve glucose homeostasis and promote growth in *Danio rerio* [22], and alleviate metabolic disorders and intestinal inflammation in *Oreochromis nilotica* [23]. Dietary butyric acid can ameliorate the negative effects of HFD on *Micropterus salmoides* growth, intestinal bacterial homeostasis, and liver health [24]. However, the role of SCFAs on muscle homeostasis and the underlying role of gut microbe in this process remains unclear in most aquatic animals.

Freshwater drum (*Aplodinotus grunniens*) is a novel aquaculture variety and is the only species that perennially inhabits in freshwater in the genus of *Aplodinotus* [25]. Freshwater drum has a promising development prospect because of its delicious flavor and high nutritional value. Therefore, we introduced freshwater drum from America and conducted extensive research. In recent years, we have overcome the artificial breeding, conducted research on feed domestication and its potential regulatory mechanisms [26], explored mechanisms of hypothermia acclimatization [27–29], and discovered specific regulatory mechanisms of adipose tissue [30], as well as the regulation of liver lipid metabolism and intestinal health by HFDs [31, 32]. However, the potential mechanism of HFD on muscle lipid metabolism through modulation of gut microbes and the metabolites SCFAs has not been revealed. Therefore, this study aimed to investigate the effects of HFD on muscle proximate composition, amino acids, long-chain fatty acids, SCFAs, and lipid metabolism, as well as the possible mechanisms by which gut microbes and metabolites SCFAs regulate gut and muscle metabolism.

## 2. Materials and Methods

### 2.1. Ethical Statement

The animal procedure was approved by the Animal Care and Use Committee of the Ethics of Nanjing Agricultural University (Nanjing, China, WXFC 2021-0006). All related procedures were performed according to the Guideline for the Care and Use of Laboratory Animals in China.

### 2.2. Experimental Design

The experiment was conducted at the Freshwater Fisheries Research Center, Chinese Academy of Fisheries Sciences (FFRC) aquaculture base. The rearing experiment was conducted as in our previous report [32]. In detail, two outdoor ponds with an area of about 667 m^2^ were selected for the HFDs experiment; each pond contained ~20,000 fish. During the rearing process, the control (Con, 6% fat) and the HFDs (12% fat) groups were fed the compound diets four times a day (6:00, 10:00, 14:00, and 18:00) until apparent satiety for 16 weeks. The feeding amount was 3%–5% of body weight every day. As the experiment was conducted in outdoor ponds, abundant planktons, microbes, and water plants in the ponds promotes the self-purification ability. Therefore, the residual baits and fish feces could be degraded, and no additional water exchange was applied except for the automatic evaporation. Throughout the rearing period, the water temperature is maintained within the optimal growth temperature for freshwater drum (22–28°C), and water quality was maintained during the aquaculture period as follows: DO > 6 mg/L, pH 7.2–7.8, NO_2_^−^ < 0.02 mg/L and NH_3_ < 0.05 mg/L. Dietary formulations are shown in Table S1.

### 2.3. Sample Collection

After the 16-week feeding experiment, the fish were starved for 24 h to eliminate the food residues in the gut. Twenty-seven fish were randomly selected from each pond and anesthetized using MS-222 (100 mg/L, Tricaine Methanesulfonate, Sigma, St. Louis, MO, USA) for sample collection. Muscle and intestine tissues (with contents) were collected on ice, immediately frozen in liquid nitrogen, and stored at −80°C for sequencing and RT-PCR analysis. The remaining muscle tissues were immediately stored at −20°C for muscle nutritional analysis.

### 2.4. Proximate Composition Analysis in Muscle

The moisture, crude protein, crude lipid, and ash contents in muscle were determined according to the standard methods of the AOAC [33]. Briefly, muscle samples were dried at 105°C to determine moisture. Crude protein was determined by the Kjeldahl method after acid digestion of the samples. Crude lipids were extracted by Soxhlet extraction with petroleum ether. Muscle ash content was determined by combustion at 550°C.

### 2.5. Nutritional Composition Analysis in Muscle

For the determination of fatty acid composition and content, muscle samples were lyophilized under vacuum, ground and pulverized, and dried. The prepared muscle samples were extracted with total fat using chloroform: methanol: water = 2:2:1, the fatty acids were methylated with potassium hydroxide-methanol, and then the fatty acid methyl esters were extracted with n-heptane. Samples were saponified, methylesterified, and analyzed by gas chromatography-mass spectrometry (Agilent 7890B-5977A, USA). Fatty acid composition and content were determined using the area normalization method of freshwater drum muscle samples.

For the determination of hydrolyzed amino acids, the method of GB/T 5009.124-2003, “Amino Acid Determination in Food,” was used for sample processing. Muscle samples were hydrolyzed with acid (6 mol/L HCl) for the determination of most amino acids by high-performance liquid chromatography (Agilent 1260, USA), and the samples were hydrolyzed with alkali (5 mol/L NaOH) for the determination of tryptophan content separately in the same machine.

For the determination of free amino acids, 10% trichloroacetic acid (TCA) was added to the muscle samples, and the supernatant was centrifuged at 2000 *g* for 20 min and adjusted to pH 2.2. Ortholeucine was used as an internal standard for quantitative determination. It was determined on a high-performance liquid chromatograph (Agilent 1260, USA).

### 2.6. SCFAs Analysis in Muscle and Intestine

Intestine and muscle samples were weighed into an Ep tube, and 400 μL of 0.5% phosphate water was added. Samples were cryo-milled (50 HZ) for 3 min, sonicated for 10 min, and centrifuged at 12,000 *g* for 15 min at 4°C. Then 200 μL of the supernatant was collected in a 1.5 mL tube, and an equal volume of *n*-butanol was added for solvent extraction. The samples were then vortexed for 10 s, sonicated at low temperature for 10 min, centrifuged at 12,000 *g* for 5 min at 4°C, and the supernatant was collected in the injection vial of the instrument (Agilent Technologies Inc., CA, USA, 8890B-5977B).

For the chromatographic conditions, HP FFAP capillary column (30 m × 0.25 mm × 0.25 μm), high purity helium (purity not less than 99.999%) as the carrier gas, flow rate of 1.0 mL/min, inlet temperature of 180°C. The injection volume was 1 μL, and the sample was injected by shunt, with a shunt ratio of 10:1 and a solvent delay of 2.5 min. The initial temperature of the column temperature chamber was 80°C, and the temperature was programed to rise to 120°C at 20°C/min, and to 160°C at 5°C/min, and then kept at 220°C for 3 min. Mass spectrometry conditions: electron bombardment ion source (EI), ion source temperature 230°C, quadrupole temperature 150°C, transmission line temperature 230°C, electron energy 70 eV. The scanning mode was selected ion scanning mode (SIM). Masshunter quantitative software (Agilent, USA, version. v10.0.707.0) was used for the automatic identification and integration of the ion fragments of SCFAs, and the detected concentration of each sample was calculated by the standard curve, which was converted to the actual content of SCFAs in the samples.

### 2.7. RNA Extraction and De Novo High-Throughput Sequencing

According to our previously described method [31], 12 muscle tissues were selected from each group, three tissues were randomly mixed, and RNA-seq was performed using four replicates. Initially, total RNA was extracted from tissue samples, and RNA concentration and purity were examined using Nanodrop2000 (ThermoFisher Ltd., Waltham, MA, USA) to construct cDNA libraries. Magnetic beads containing oligo (dT) for A-T base pairing with ployA were used to isolate mRNA from total RNA. The mRNA was then randomly split into small fragments of about 300 bp, and stable double-stranded structures were synthesized using random primers. The sticky ends of the double-stranded cDNA were patched to flat ends, followed by the addition of the “A” tail to the 3' end, product purification, and fragment sorting, followed by PCR amplification to obtain the final library. Finally, de novo high-throughput sequencing was performed using Illumina NovaSeq6000 (Majorbio Bio-pharm Technology Co., Ltd., Shang, China). The final validated data were obtained through sequencing data quality control, sequence comparison analysis, and transcript assembly.

### 2.8. Intestine Microbiota Analysis

The methods for the detection of gut microbes have been described in our previous paper [32]. The general method is as follows: extract gut microbial DNA, followed by checking DNA integrity. PCR amplification was performed using specific primers. After purification and recovery, quantification was performed, and finally, the purified PCR products were sequenced using the MiSeq platform (Illumina, San Diego, CA, USA). Reads were spliced, controlled, and checked for sequence quality to obtain final valid data.

### 2.9. Real-Time PCR

Expression levels of key genes involved in lipid and glucose metabolism were determined by RT-PCR. Briefly, the RNAiso-Plus reagent (Takara Co., Ltd., Dalian, China) was used to extract total RNA from muscle and gut tissues according to the manufacturer's instructions. RNA was reverse transcribed into cDNA using RT-PCR kits (Takara Co., Ltd., Dalian, China). Finally, RT-PCR was performed with SYBR Green (Takara, Dalian, China) on a Takara 800 rapid real-time PCR system. The expression level of *β*-actin remained stable under different treatments and, therefore, was used as an internal reference gene to normalize the CT value of the target gene. All primer sequences were obtained based on the transcriptome sequencing of freshwater drum (Table 1). All primers were produced by Shanghai Generay Biotechnology Co., Ltd., Shanghai, China.

### 2.10. Statistical Analysis

Data were analyzed using SPSS software (version 26.0) and expressed as mean ± standard error of the mean (SEM). Gene expression levels were calculated by 2^−*ΔΔct*^. Statistical differences between the two groups were analyzed by Student's *t*-test. Pearson correlation analysis was used to evaluate the correlation between different indicators. Significant differences were as follows: *⁣*^*∗*^ represents *p* < 0.05 and *⁣*^*∗∗*^ represents *p* < 0.01.

## 3. Results

### 3.1. Acetic Acid Involves Muscular Lipid Metabolism in *A. grunniens* Under HFD

**Figure 1:**
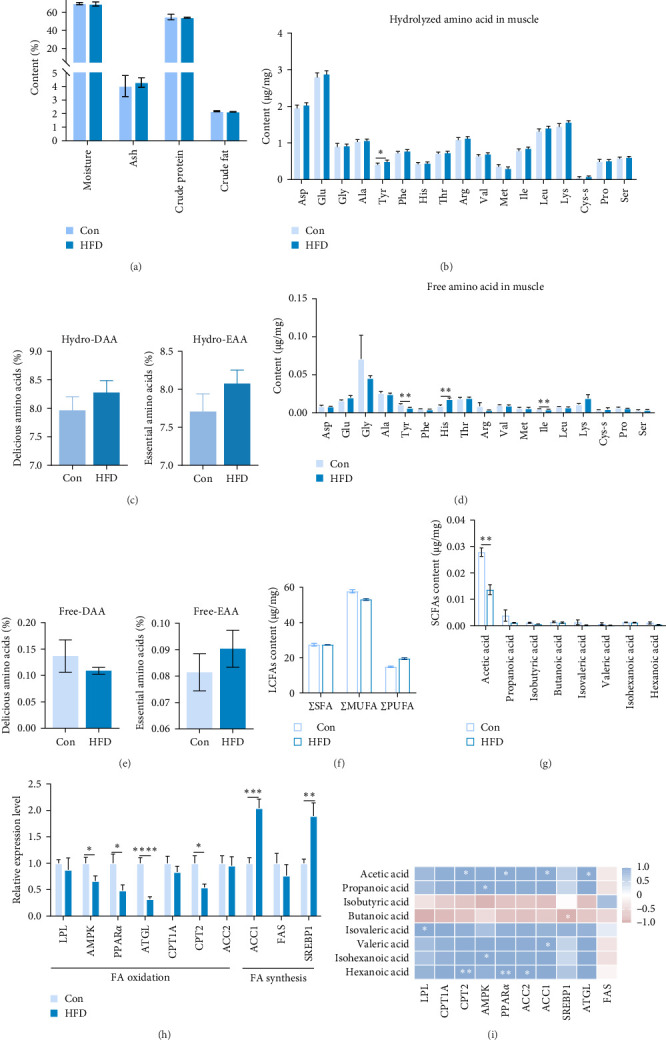
Acetic acid involves muscular lipid metabolism in freshwater drum under high-fat diet (HFD). (A) Muscular proximate composition. (B,C) Hydrolyzed amino acids. (D,E) free amino acids: DAA, delicious amino acid; EAA, essential amino acid. (F) Fatty acids content: ∑MUFA, total monounsaturated fatty acid; ∑PUFA, total polyunsaturated fatty acid; ∑SFA, total saturated fatty acid. (G) Short-chain fatty acids (SCFAs) content in muscle. (H) Lipid metabolism-related genes in muscle. (I) Pearson correlation analysis of SCFAs and lipid metabolism genes. *⁣*^*∗*^Represents a significant difference or correlation. *⁣*^*∗*^*p* < 0.05, *⁣*^*∗∗*^*p* < 0.01, *⁣*^*∗∗∗*^*p* < 0.001, and *⁣*^*∗∗∗∗*^*p* < 0.0001. Results were indicated as mean ± SEM. (A–G) *n* = 6. (H) *n* = 9.

To investigate the effects of HFD on nutrient composition and lipid metabolism in the muscle of freshwater drum, we first assessed the proximate composition as well as the amino acid and fatty acid composition in the muscle. Results indicate no significant differences were observed in moisture, ash, crude protein, and crude fat contents between HFD and Con (Figure 1A). Free amino acid assay reveals tyrosine (Tyr) was significantly increased in HFD (*p* < 0.05, Figure 1B). Meanwhile, hydrolyzed amino acids histidine (His) was significantly increased, Tyr and isoleucine (Ile) were markedly decreased (*p* < 0.01, Figure 1D). However, delicious amino acids (DAAs) and EAAs exhibit no statistical difference, both in free and hydrolyzed amino acids (*p* > 0.05, Figure 1C,E). Interestingly, we found long-chain fatty acid contents, including total saturated fatty acids (∑SFAs), total monounsaturated fatty acids (∑MUFAs), and total polyunsaturated fatty acids (∑PUFAs), also exhibit no statistical difference (*p* > 0.05, Figure 1F) between HFD and Con groups. For SCFAs, acetic acid was significantly reduced (*p* < 0.01), whereas the other SCFAs (propanoic acid, isobutyric acid, butanoic acid, isovaleric acid, valeric acid, isohexanoic acid, and hexanoic acid) did not show significant changes (*p* > 0.05, Figure 1G).

We next analyzed the effects of HFD on lipid metabolism in the muscle, the expression levels of fatty acid oxidation-related genes AMP-activated protein kinase (AMPK), peroxisome proliferator-activated receptor alpha (PPAR*α*), adipose triglyceride lipase (ATGL), and carnitine palmitoyl transferase 2 (CPT2) were remarkably decreased in HFD (*p* < 0.05), while fatty acid synthesis-related genes sterol regulatory element binding protein-1 (SREBP1) and acetyl-CoA carboxylase alpha (ACC1) were markedly increased in HFD (*p* < 0.05, Figure 1H). However, the expression levels of lipoprotein lipase (LPL), fatty acid synthase (FAS), carnitine palmitoyl transferase 1A (CPT1A), and acetyl-CoA carboxylase beta (ACC2) were not statistically different (*p* > 0.05). Moreover, Pearson correlation analysis reveals acetic acid was significantly correlated with CPT2, PPAR*α*, ACC1, and ATGL (*p* < 0.05, Figure 1I). These results indicate acetic acid correlates with fatty acid oxidation and synthesis under HFD administration.

### 3.2. Transcriptome Analysis Reveal HFD Affects Muscular Glucose Metabolism and Lipid Metabolism in *A. grunniens*

**Figure 2:**
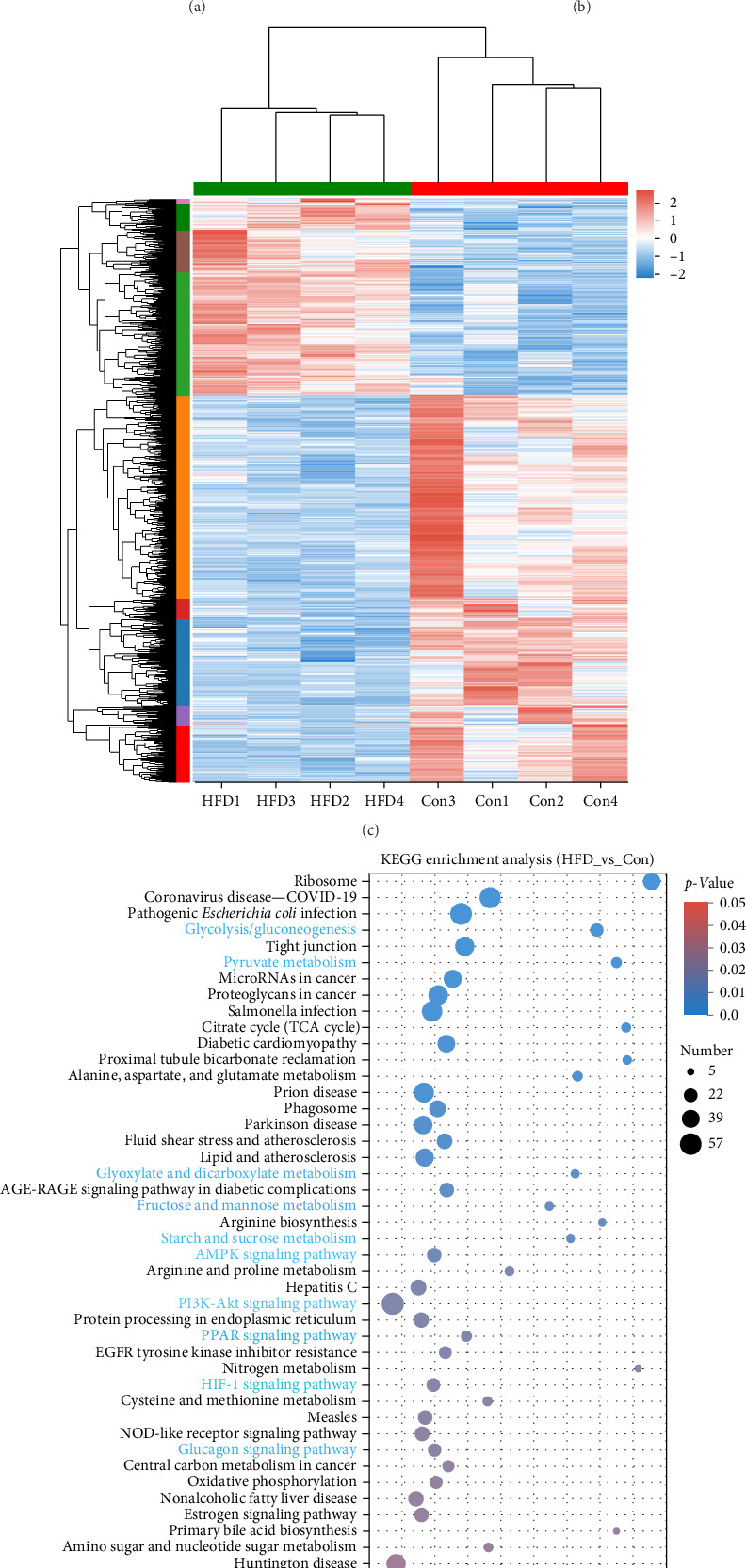
Transcriptome analysis reveals high-fat diet (HFD) affects muscular glucose metabolism and lipid metabolism in freshwater drum. (A) Venn diagram analysis. (B) Volcano plots of expression difference. (C) Cluster heatmap of differentially expressed genes (DEGs). (D) KEGG enrichment of DEGs.

To further understand the underlying molecular mechanisms of HFD in the muscle, transcriptome sequencing was performed. The results showed that 12,206 genes were annotated in Con and 11,797 in HFD, of which 11,282 genes were co-enriched (Figure 2A). Among them, a total of 1901 differentially expressed genes (DEGs) were identified (|fold change| ≥ 2, *p*-adjust < 0.05), of which 642 were significantly upregulated, and 1259 were downregulated in HFD compared to Con (Figure 2B). These DEGs were clustered into different subgroups in the heatmap based on their expression levels (Figure 2C). KEGG enrichment reveals DEGs were enriched into 54 differential pathway items (Figure 2D), of which were mainly enriched in energy metabolism, including glucose metabolism and lipid metabolism (glycolysis/gluconeogenesis, pyruvate metabolism, glyoxylate and dicarboxylate metabolism, fructose and mannose metabolism, starch and sucrose metabolism, AMPK signaling pathway, PI3K-Akt signaling pathway, PPAR signaling pathway, HIF-1 signaling pathway, glucagon signaling pathway). The transcriptome results illustrate that HFD affects muscle glucose metabolism and lipid metabolism.

### 3.3. Acetic Acid Implicates Glycolysis-Regulated Lipid Metabolism in the Muscle of *A. grunniens* Under HFD

**Figure 3:**
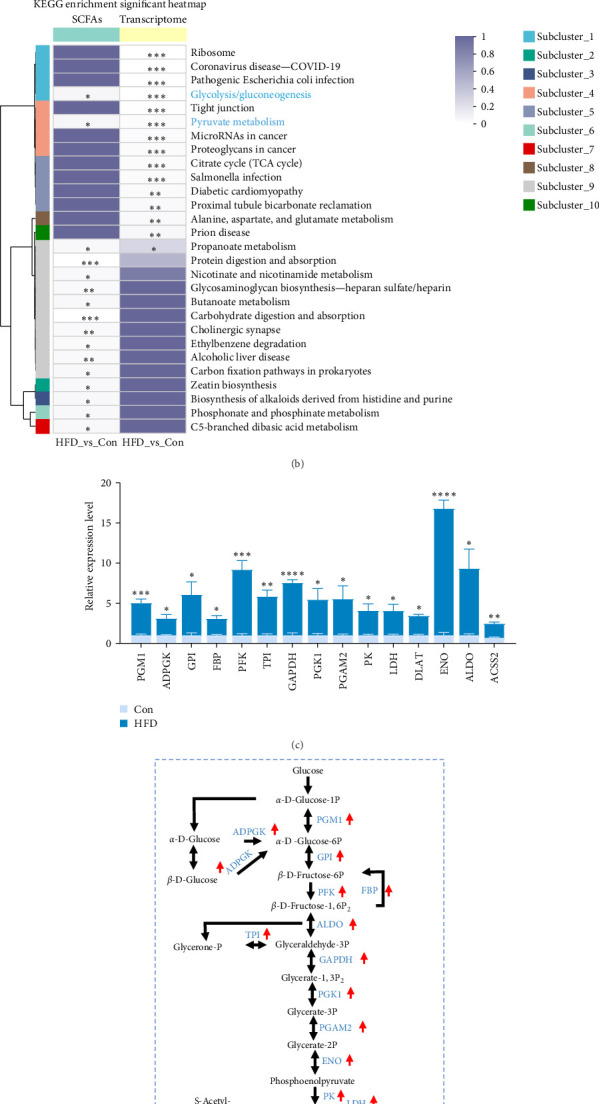
Acetic acid implicates glycolysis-regulated muscular lipid metabolism in freshwater drum under high-fat diet (HFD). (A,B) Transcriptome and short-chain fatty acid (SCFA) association analysis in the muscle. (C) Differential gene expression in the enrichment pathway. (D) Relationship between differential genes and acetic acid regulation. (E) Pearson correlation analysis of glucose metabolism and lipid metabolism-related genes. *⁣*^*∗*^Represents a significant differenceor correlation. *⁣*^*∗*^*p* < 0.05, *⁣*^*∗∗*^*p* < 0.01, *⁣*^*∗∗∗*^*p* < 0.001, and *⁣*^*∗∗∗∗*^*p* < 0.0001. Results were indicated as mean ± standard error of the mean (SEM), *n* = 9.

Based on the above findings, we next performed an integrated analysis of transcriptome and SCFAs to further explore the pathway of acetic acid involved in muscle lipid metabolism. As expected, six metabolic pathways were correlated with acetic acid (Figure 3A), including pyruvate metabolism, glycolysis/gluconeogenesis, glycosaminoglycan biosynthesis-heparan sulfate/heparin, cholinergic synapse, carbohydrate digestion and absorption, protein digestion and absorption. Specifically, glycolysis/gluconeogenesis and pyruvate metabolism were significantly correlated with acetic acid (Figure 3B). Importantly, the expression of glycolysis-related genes was upregulated in these two pathways (*p* < 0.05, Figure 3C), and acetic acid was implicated in regulating the glycolytic pathway (Figure 3D). Therefore, we hypothesize that reduced acetic acid in muscle may be associated with enhanced glycolysis. Then, glycolysis and lipid metabolism-related genes were performed by Pearson correlation analysis. The results showed that key genes of glycolysis were significantly correlated with CPT2, AMPK, ACC2, and FAS in HFD (*p* < 0.05, Figure 3E). These results indicate acetic acid may regulate lipid metabolism by glycolysis in freshwater drum under HFD.

### 3.4. Acetic Acid Involves Intestinal Lipid Metabolism in *A. grunniens* Under HFD

**Figure 4:**
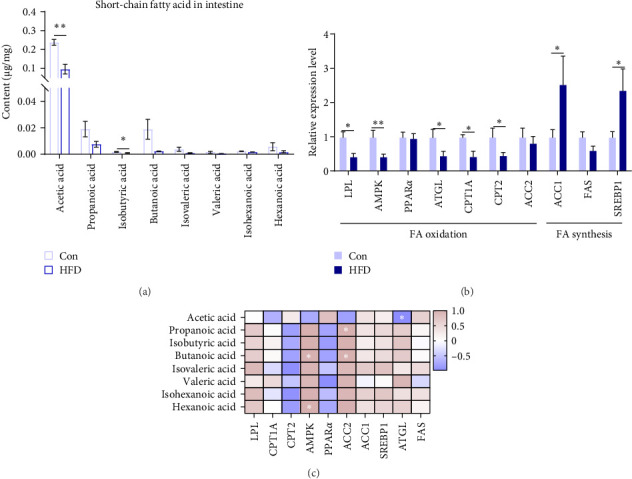
Acetic acid involves intestinal lipid metabolism of the freshwater drum under high-fat diet (HFD). (A) Short-chain fatty acids (SCFAs) in the intestine. (B) Lipid metabolism-related genes in the intestine. (C) Pearson correlation analysis of SCFAs and lipid metabolism genes. *⁣*^*∗*^Represents a significant difference or correlation. *⁣*^*∗*^*p* < 0.05 and *⁣*^*∗∗*^*p* < 0.01. Results were indicated as mean ± standard error of the mean (SEM). (A) *n* = 6. (B) *n* = 9.

To further clarify whether changes in muscle acetic acid content are regulated by the intestine, we further examined intestinal SCFAs content. The results indicated that intestinal acetic acid and isobutyric acid content was remarkably reduced (*p* < 0.05), while there were no significant differential changes in the content of other SCFAs, including propanoic acid, butanoic acid, isovaleric acid, valeric acid, isohexanoic acid, hexanoic acid (*p* > 0.05, Figure 4A). This result is consistent with the significant reduction of acetic acid content in muscle. Similarly, we examined the expression levels of intestinal lipid metabolism genes, fatty acid synthesis-related SREBP1 and ACC1 were significantly upregulated (*p* < 0.05), while fatty acid oxidation-related LPL, AMPK, ATGL, CPT1A, and CPT2 were notably downregulated (*p* < 0.05, Figure 4B). Meanwhile, Pearson correlation analysis of intestinal SCFAs with lipid metabolism genes reveals ATGL was significantly correlated with intestinal acetic acid (*p* < 0.05, Figure 4C). These results reveal acetic acid involves in intestinal lipid metabolism.

### 3.5. Acetic Acid-Related Intestine Microbes Affect Muscular and Intestinal Glucose Metabolism and Lipid Metabolism in *A. grunniens* Under HFD

**Figure 5:**
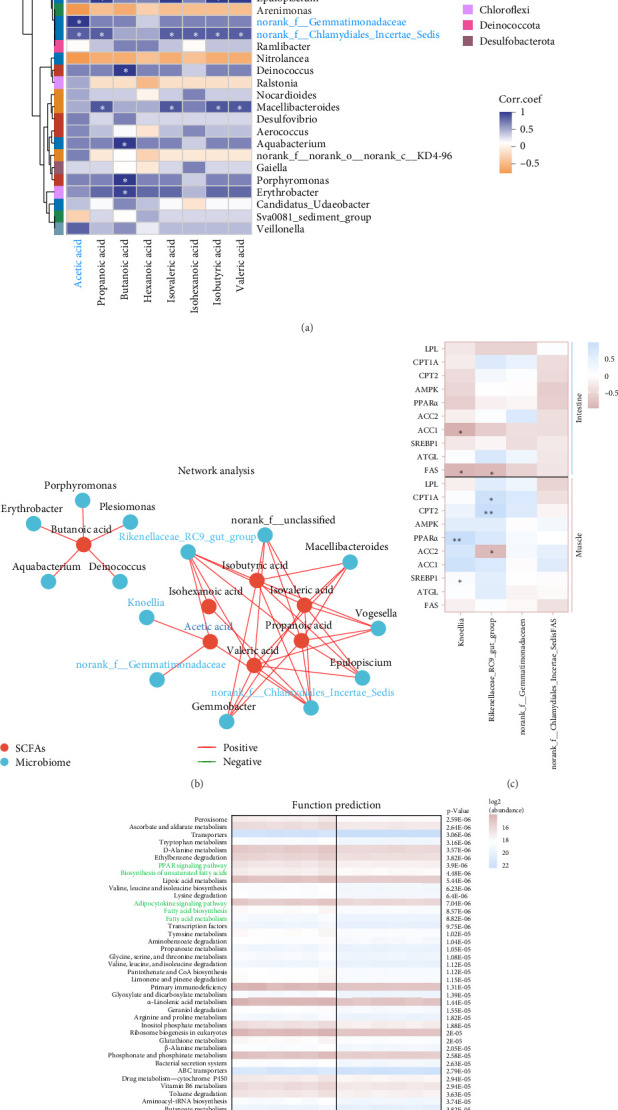
Acetic acid-related intestinal microbes affect muscular and intestinal glucose metabolism and lipid metabolism in freshwater drum under high-fat diet (HFD). (A,B) Intestinal short-chain fatty acids (SCFAs) and differential microbial association analysis. (C) Pearson correlation analysis of key microbes with intestinal and muscle lipid metabolism-related genes. (D) KEGG pathway abundance composition analysis of intestine microbes. *⁣*^*∗*^Represents a significant difference or correlation. *⁣*^*∗*^*p* < 0.05 and *⁣*^*∗∗*^*p* < 0.01.

It is clear that acetic acid is a metabolite of gut microbes; we next integrated SCFAs and microbes to identify the key microbes that regulate acetic acid. Results reveal *Rikenellaceae_RC9_gut_group*, *Knoellia*, *norank_f__Gemmatimonadaceae* and *norank_f__Chlamydiales_Incertae_Sedis* were significantly correlated with acetic acid (*p* < 0.05, Figure 5A,B). Furthermore, *Rikenellaceae_RC9_gut_group* and *Knoellia* correlate with lipid metabolism in different manners between the intestine and muscle (Figure 5C). Specifically, *Rikenellaceae_RC9_gut_group* was remarkably associated with ACC1 and FAS (*p* < 0.05), and *Knoellia* was significantly correlated with FAS (*p* < 0.05) in the intestine. However, *Rikenellaceae_RC9_gut_group* was markedly associated with PPAR*α* (*p* < 0.01), and *Knoellia* was significantly correlated with CPT1A, CPT2 and ACC2 (*p* < 0.05). Furthermore, functional prediction of intestinal microbes with KEGG enrichment reveals gut microbes mainly involved in glucose metabolism and lipid metabolism (Figure 5D), including the PPAR signaling pathway, biosynthesis of unsaturated fatty acids, adipocytokine signaling pathway, fatty acid biosynthesis, fatty acid metabolism, pyruvate metabolism, and lipid biosynthesis proteins. Among them, PPAR signaling and pyruvate metabolism were also significantly enriched in the muscle transcriptome. Therefore, acetic acid-mediated lipid and glucose metabolism in the muscle and intestine were proved to be impacted by intestinal microbes.

### 3.6. Hypothesized Regulatory Mechanisms of Gut Microbes on Muscle in *A. grunniens* Under HFD

**Figure 6:**
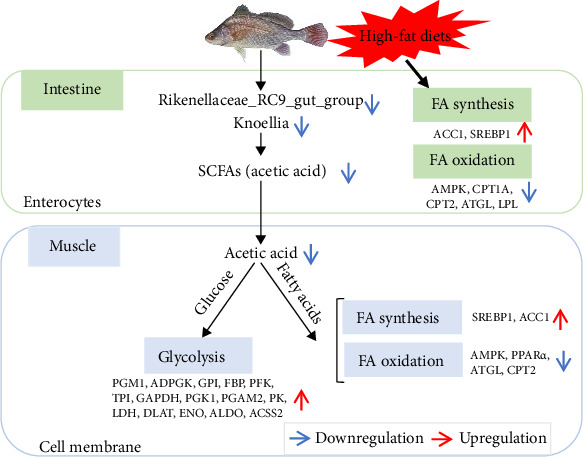
Hypothesized regulatory mechanisms of gut microbes on muscle in freshwater drum under high-fat diet (HFD).

Based on the above studies, we provide possible regulatory mechanisms of intestine microbes and SCFAs on muscular metabolism under HFD (Figure 6). HFD administration promotes fatty acid synthesis and inhibits fatty acid oxidation in the intestine. Additionally, the decreased abundance of the intestine microbes *Rikenellaceae_RC9_gut_group* and *Knoellia* reduce acetic acid levels. Acetic acid could transfer from intestinal epithelium into muscle cells, thereby regulating the rate of glycolysis and participating in the *β*-oxidation of fatty acids. Taken together, the acetic acid produced by gut microbe *Rikenellaceae_RC9_gut_group* and *Knoellia* can regulate muscle glucose metabolism and lipid metabolism under HFD, providing insights for future studies of the intestine-muscle axis in freshwater drum.

HFDs affect levels of the microbial metabolite acetic acid by altering the abundance of gut microbes, which in turn affects muscle glucose and lipid metabolism. Red arrows indicate upregulation of gene expression levels, and blue arrows indicate microbial abundance or downregulation of gene expression levels.

## 4. Discussion

To improve economic efficiency, HFD is inevitably and widely used in aquaculture. However, HFD has several negative effects and has become one of the challenges in the aquaculture industry [34]. Our previous studies have demonstrated that HFD causes liver fat deposition, oxidative stress, and lipid metabolism disorders in freshwater drum [31]. In addition, HFD decreases the abundance of gut microbes, inducing immune and inflammatory responses in the gut [32]. However, there is still confusion about the effects of HFD on muscle metabolism. Therefore, the effects of HFD on muscle metabolism were investigated in this study to reveal the potential regulatory mechanisms.

### 4.1. Acetic Acid Regulates Muscle Lipid Metabolism in Freshwater Drum

It is well known that HFD induces fat accumulation in animals, particularly in the liver, abdomen, adipose tissue, and muscle [5]. In muscle, fat content correlates favorably with dietary fat, as reported in a previous study [35]. However, in the current study, 16 weeks of HFD feeding had no significant effect on muscle crude fat. In addition, the muscle moisture, ash, crude protein content, total content of free amino acids, hydrolyzed amino acids, and long-chain fatty acids also showed insignificant differences. Surprisingly, our results are in contrast to others [36–38]. This may be because fat tolerance differs between species and that 12% dietary lipid does not alter the nutrient composition of freshwater drum muscle. Previous studies have shown that increasing acetic acid levels in the diet reduces the fat deposition in the liver of *O. nilotica* but does not affect muscle lipid metabolism [39, 40]. Contrary studies show that increased dietary acetic acid leads to increased lipid deposition in *O. nilotica* [41]. An interesting result found in the present study is that HFD dramatically decreased acetic acid content. Thus, this shows that there is a potential crosstalk between acetic acid and lipids. We found that HFD inhibited AMPK, PPAR*α*, ATGL, and CPT2 and activated SREBP1 and ACC1, suggesting that HFD inhibited muscle lipolysis and promoted adipogenesis in freshwater drum. These results are in line with previous reports [42, 43]. More importantly, acetic acid is involved in the regulation of CPT2, PPAR*α*, ACC1, and ATGL transcription levels. Among them, PPAR*α* and CPT2 are key genes regulating fatty acid *β*-oxidation, while ACC1 is mainly involved in lipid synthesis [44–46]. From the above results, we conjectured that acetic acid is involved in the regulation of muscle lipid metabolism in freshwater drum.

### 4.2. Glycolysis Involves Acetic Acid-Mediated Muscular Lipid Metabolism in Freshwater Drum Under HFDs

In light of the above studies, we further analyzed the potential regulatory mechanisms of HFD on muscle metabolism by RNA-seq. Among the 54 pathways significantly enriched in DEGs, glucose and lipid metabolism-related were the most prominent, suggesting that HFD affects muscle glucose metabolism and lipid metabolism. This result is similar to that of high-fat fed *Epinephelus fuscoguttatus* ♀ × *E. lanceolatus* ♂ [47]. Impressively, significantly correlated acetic acid with glycolytic metabolism (glycolysis/glycolysis, pyruvate metabolism) pathway, and upregulation of the genes in these pathways illustrates that HFD-induced decrease in muscle acetic acid is associated with an increase in glycolysis. Not surprisingly, similar studies have demonstrated that dietary acetic acid inhibits glucose catabolism in *O. nilotica* [41]. In addition, studies have demonstrated that fish pyruvate produced by glycolytically can be converted to acetyl-coenzyme A (Ac-CoA), which directly participates in the TCA cycle for energy supply and de novo lipid synthesis [48]. In particular, Acss2 is a critical enzyme catalyzing the synthesis of Ac-CoA from acetic acid in mitochondria [49]. Most genes related to glycolysis are significantly associated with FAS, and the ACSS2 gene is significantly associated with CPT2, AMPK, and ACC2, indicating that glycolysis and ACSS2-mediated energy metabolism in muscle may be involved in muscle lipid metabolism by regulating Ac-CoA, but this is an aspect that needs to be explored in further studies.

### 4.3. Gut Microbes Regulate Muscle and Gut Lipid Metabolism by Acetic Acid

A number of studies have demonstrated that the composition of gut microbes affects lipid accumulation and expression of key genes involved in lipid metabolism [50, 51]. Consequently, we further explored whether intestine microbes regulate intestinal lipid metabolism by influencing the metabolism of acetic acid. Significantly reduced acetic acid levels, remarkably upregulation of SREBP1 and ACC1 expression, and downregulation of LPL, AMPK, ATGL, CPT1A, and CPT2 expression demonstrated that HFD inhibits intestinal lipolysis and promotes adipogenesis in freshwater drum. Importantly, intestinal acetic acid was significantly correlated with ATGL, which is consistent with findings in muscle. In addition, the gut microbes *Rikenellaceae_RC9_gut_group*, *Knoellia*, *norank_f__Gemmatimonadaceae*, *norank_f__Chlamydiales_Incertae_Sedis* may be involved in the regulation of acetic acid metabolism. Among them, *Rikenellaceae_RC9_gut_group* and *Knoellia*, significantly correlated with decreased acetic acid, were recognized to participate in lipid metabolism in the intestine and muscle. This is similar to a previous study that reduced levels of acetic acid in mammals are associated with a reduction in the abundance of *Rikenellaceae_RC9_gut_group* [52]. In addition, the *Rikenellaceae_RC9_gut_group* can be affected by diet composition and external environment in aquatic animals [53, 54] and may be involved in the metabolism of fatty acids and may have a negative effect on muscle quality [55]. However, its function remains to be further investigated. Studies on *Knoellia* have found possible involvement in glucose metabolism [56], but there is a paucity of reports in aquatic animals. Concurrently, significant enrichment of glucose metabolism and lipid metabolism pathways was found by functional prediction of differential microbes, which further proved our conjecture. Furthermore, in mammals, SCFAs have been shown to directly regulate muscle metabolism through the gut-muscle axis [57]. However, as there is no public bacterial strain of *Rikenellaceae_RC9_gut_group* and *Knoellia*, we also have not successfully isolated and cultured these two microbes from the intestine of freshwater drum, the underlying mechanism of these two microbes on muscle physiology and homeostasis under HFD still remain further investigation. In summary, our findings uncovered that HFD may influence intestine and muscle acetic acid levels by influencing intestine microbes (*Rikenellaceae_RC9_gut_group* and *Knoellia*), thereby modulating muscle glucose metabolism and lipid metabolism.

## 5. Conclusions

In conclusion, 12% HFD administration did not affect muscle proximate composition, total amino acids, and fatty acids levels in the freshwater drum but reduced muscle and intestine acetic acid levels. Importantly, acetic acid-mediated lipid and glucose metabolism in the muscle and intestine were proved to be impacted by intestinal microbes (*Rikenellaceae_RC9_gut_group* and *Knoellia*). These results provide a rational basis for HFD on muscle metabolism in freshwater drum.

## Figures and Tables

**Table 1:** Primer sequences of target genes for RT-PCR analysis.

Accession no.	Gene	Primer sequence (5′—3′)	Amplification efficiency (%)	Amplification size (bp)	Annealing temperature (°C)
XM_027277534.1	*β*-Actin	F: AGGCTGTGCTGTCCCTGTAT R: CTGTGGTGGTGAAGGAGTAG	102.08	202	55

XM_019262525.2	LPL	F: CAGCCGTGCAGTATGTGACT R: AGGTTTTGGAGGTGCTGTTG	95.84	228	55

XM_010738413.3	AMPK	F: TCCCTCCTACAGCAACAAC R: GACGCCAGGTAGAAATCC	104.48	192	60

XM_010749024.2	PPAR*α*	F: GTGCCTCTCTGTGGGAATGT R: GCTTCGTGGATCTGCCTTAC	106.38	159	55

XM_010741340.2	ATGL	F: ACGGGGAGAACATACTGGTG R: TGGAAGCTGGTGGAGTTGT	97.49	242	60

XM_010741011.3	FAS	F: TGGCATCGAGTACAACAAGC R: TTGGCACGAAGTAGCATCAC	95.93	219	55

XM_010730705.3	SREBP1	F: TTCCTCTCCCTCAACCCTCT R: TTACGGGCTCTCCATACACC	103.39	235	55

XM_019273008.1	CPT1A	F: TCAGAGGCAGGAGCCCTATT R: GTGCATGTTCACCACGTTCC	99.26	153	60

XM_010734381.3	CPT2	F: TGACGAGAAAGGGCGACACC R: CATCCCTGTTCTCACTGGTC	98.27	185	60

XM_018668480.2	ACC2	F: AGAGGACCATCCGTTTTGTG R: TTCAGAGGAATGACCCCATC	105.38	200	55

XM_019271858.1	ACC1	F: CTGGAGGAGACGGTGAAAAG R: TGCGTATCTGCTTGAGGATG	106.46	247	60

XM_010730931.3	PGM1	F: GTGTGGAGAGGAGAGCTTCG R: CACTCTGTTTCCTGGTGGCT	101.38	107	60

XM_010744093.3	ADPGK	F: GGAGGTGACTGATGGGTTGG R: GCATAGAGTCAGGCCACCTC	97.65	202	60

XM_010733121.2	GPI	F: GCCATTGCCCTGCATATTGG R: AAGTAGGCAGTGAAGCGGT	104.53	210	55

XM_010747969.3	FBP	F: TTACGTTGGACCCTGCCATC R: GGCAATGGTGCGGTGAAT	96.28	223	55

XM_027278805.1	PFK	F: GGCCTTGTGTTTCTTCCCTCA R: TCTCCCAAGTAGCAGGACGG	98.76	256	55

XM_010747789.3	TPI	F: TTGACGGTTTCCTTGTGGGT R: GCCGGGTTTCTTATGCCTTG	103.72	81	50

XM_010743420.3	GAPDH	F: CATCTTTGATGCTGGCGCTG R: GCCGGGTTTCTTATGCCTTG	108.62	108	55

XM_019265778.2	PGK1	F: AACTTCCCATCAGCCCACTG R: GGTTCGTTCTTTTGCACGCT	97.73	147	60

XM_010732246.3	PGAM2	F: AGACGGGCCATATTCAGCAC R: AAGGGCTGAGTGATGTGTGG	96.51	121	60

XM_010757249.3	PK	F: AATAAAAACTGTCGCCCCAGC R: AAGGGCTGAGTGATGTGTGG	103.93	215	55

XM_010757306.3	LDH	F: TGGAGGACGGATTTACACTCA R: CGCCCACCACTGTCACTTTAT	106.41	141	55

XM_010744559.3	DLAT	F: CCAGGGAGGCACTTTCACAA R: CGCCCACCACTGTCACTTTAT	98.61	299	60

XM_027285787.1	ENO	F: GCCGAGTGGGTAAACATGAC R: AGGTACTGTCTCTGTGCCCTT	105.86	219	60

XM_019260493.2	ALDO	F: CTATTTTGGGCCTGGGGACA R: CACTGAGGGTCTTCTCGCAG	98.59	255	55

XM_027284085.1	ACSS2	F: CCCTGATCCAGTTGGTGAGC R: GGACAATCTCTGCGTCTGGG	105.93	161	55

*Note:* The mRNA sequences for each gene were obtained from the freshwater drum transcriptome sequencing database.

## Data Availability

The data that support the findings of this study are available from the corresponding author upon reasonable request.

## Supporting Information

Supporting Information Table S1: Formulation and proximate composition of experimental diets.
