# Supplementary material for: Gut Microbe Rikenellaceae_RC9_gut_group and Knoellia-Mediated Acetic Acid Regulates Glucose and Lipid Metabolism in the Muscle of Freshwater Drum (Aplodinotus grunniens) Under High-Fat Diets
Source: Aquac Nutr. 2025 Apr 16;2025:9667909. doi: 10.1155/anu/9667909 (PMC12017940; doi:10.1155/anu/9667909)
Supplement: Supporting Information — Table S1: Formulation and proximate composition of experimental diets. [file 9667909.f1.docx]

Table S1 Formulation and proximate composition of experimental diets

| Ingredient/% | 6%(Con) | 12%(HFD) | Nutriton value (%, dry matter) | 6%(Con) | 12%(HFD) |
| --- | --- | --- | --- | --- | --- |
| fish meal | 55 | 55 | Dry matter, DM | 87.001 | 88.216 |
| vital wheat gluten | 4 | 4 | Crude protein, CP | 45.131 | 45.131 |
| soybean protein concentrated | 6 | 6 | Ether extract, EE | 6.07 | 12.048 |
| α-starch | 18.5 | 5 | Nitrogen Free Extract, NFE | 6.513 | 6.5435 |
| carboxymethylcellulose | 0 | 7.4 | Crude fat, CF | 0.598 | 0.598 |
| fish oil | 1 | 7.1 | Ash | 14.275 | 14.3055 |
| Saccharomyces cerevisiae | 3 | 3 | Ca | 3.2861 | 3.2861 |
| squid meal | 2 | 2 | P | 2.16595 | 2.1646 |
| sodium humate | 2 | 2 | Total P | 2.12715 | 2.1231 |
| Sodium butyrate | 1 | 1 | Lysine | 3.1891 | 3.1891 |
| Ethoxyquin | 0.5 | 0.5 | Methionine+Cysteine | 1.188 | 1.188 |
| choline chloride(50%) | 1 | 1 | Methionine | 1.1874 | 1.1874 |
| vitamin premix^a^ | 1 | 1 | Threonine | 1.7298 | 1.7298 |
| mineral premix^b^ | 1 | 1 | Arginine | 2.5318 | 2.5318 |
| calcium dihydrogen phosphate | 2 | 2 | Fe | 203.27 | 203.27 |
| Attapulgite | 2 | 2 | Gross Energy^c^ | 15.702 | 15.72444 |
| total | 100 | 100 |  |  |  |

Note: ^a^ Mineral contents per kg diets: FeSO_4_⋅7H_2_O, 250 mg; CuSO_4_⋅5H_2_O, 20 mg; ZnSO_4_⋅7H_2_O, 220 mg; Na_2_SeO_3_, 0.4 mg; MnSO_4_⋅4H_2_O, 70 mg; CoCl_2_⋅6H_2_O, 1 mg; KI, 0.26 mg.

^b^ Vitamin contents per kg diets:Vitamin A, 9000 IU; Vitamin B1, 3.2 mg; Vitamin B2, 10.9 mg; Vitamin B5, 20 mg; Vitamin B6, 5 mg; Vitamin B12, 0.016 mg; Vitamin C, 50 mg; Vitamin D, 2000 IU; Vitamin E, 45 mg; Vitamin K3, 2.2 mg; Niacin, 28 mg; Folic acid, 1.65 mg; Pantothenate, 10 mg; Choline, 600 mg.

^c^ Energy, calculated by using standard physiological fuel values of 37.7, 16.7, and 16.7 kJ / g for protein, lipid and carbohydrate, respectively.^[[1]](#footnote-1)^

1. All ingredients are provided by fish disease and nutrition department of Freshwater Fisheries Research Center of Chinese Academy of Fishery Sciences. [↑](#footnote-ref-1)
